# An acceleration in hypertension-related mortality for middle-aged and older Americans, 1999-2016: An observational study

**DOI:** 10.1371/journal.pone.0225207

**Published:** 2020-01-15

**Authors:** Steven J. Forrester, Elena V. Dolmatova, Kathy K. Griendling

**Affiliations:** Department of Medicine, Division of Cardiology, Emory University, Atlanta, Georgia, United States of America; International University of Health and Welfare, School of Medicine, JAPAN

## Abstract

**Background:**

Hypertension-related mortality has been increasing in recent years; however, limited information exists concerning rate, temporal, secular, and geographic trends in the United States.

**Methods and results:**

Using CDC death certificate data spanning 1999–2016, we sought to delineate trends in deaths attributable to an underlying cause of hypertension using joinpoint regression and proportion testing. From 1999–2016, the hypertension-related mortality rate increased by 36.4% with an average annual percent change (AAPC) of 1.8% for individuals ≥ 35 years of age. Interestingly, there was a notable acceleration in the AAPC of hypertension mortality between 2011 and 2016 (2.7% per year). This increase was due to a significant uptick in mortality for individuals ≥ 55 years of age with the greatest AAPC occurring in individuals 55–64 (4.5%) and 65–74 (5.1%) years of age. Increased mortality and AAPC were pervasive throughout sex, ethnicity, and White and American Indian or Alaska Native race, but not Black or African American race. From 2011–2016, there were significant increases in AAPC for hypertension-related mortality with contributing causes of atrial fibrillation, heart failure, diabetes, obesity, and vascular dementia. Elevated mortality was observed for conditions with a contributing cause of hypertension that included chronic obstructive pulmonary disease, diabetes, Alzheimer’s, Parkinson’s, and all types of falls. Geographically, increases in AAPCs and mortality rates were observed for 25/51 States between 2011 and 2016.

**Conclusions:**

Our results indicate hypertension-related mortality may have accelerated since 2011 for middle-aged and older Americans, which may create new challenges in care and healthcare planning.

## Introduction

Hypertension is a global health problem that carries significant health and economic ramifications. Current estimates indicate 34% of U.S. adults 20 years or older—more than 85 million people—suffer from this “silent killer” [1]. Importantly, hypertension is a contributing factor in the development of cardiovascular [1, 2], metabolic [3], renal [4], pulmonary, and neurological diseases/complications [5] that carry high mortality rates, especially for older individuals [6]. The significantly high prevalence and far reaching influence on various disease states means that hypertension is a large financial burden on the health care system, which is predicted to exceed $220 billion dollars by 2035 (2013–2014 estimate was $53 billion dollars) [1].

Timely detection and treatment of hypertension are considered to be effective in reducing cardiovascular events, deaths, and healthcare costs [7]. Over the next decade, the US will see a significant portion of the population move into the age groups that are associated with the highest levels of hypertension-related mortality. Thus, resources and preventative efforts will need to be targeted and allocated effectively. However, apart from prevalence data, information on national, demographic, and geographic trends in hypertensive-related mortality and its comorbidities remains limited. Special attention must be given to these trends given that the severity and consequences of hypertension vary by age, sex, race, and geographical area. Understanding these trends will help to effectively distribute resources and focus clinical care. Using the Centers for Disease Control and Prevention’s Wide-ranging Online Data for Epidemiologic Research (CDC WONDER) database, this study aims to provide up-to-date trend analysis for hypertension-related mortality and highlights a growing and urgent clinical and economic problem.

## Methods

### Data collection and characteristics

All data were acquired from the CDC WONDER database (wonder.cdc.gov) in collaboration with the National Center for Health Statistics and the Vital Statistics Cooperative Program [8] using detailed mortality data on underlying cause of death between 1999–2016. Underlying cause of death, according to the U.S. Department of Health and Human Services, is defined as the disease or injury that initiated the chain of events that directly led to death [9]. For instance, acute myocardial infarction may be listed as an immediate event, atherosclerotic coronary artery disease listed as an underlying cause with an interval onset of 2 years, and hypertension listed as an underlying cause with an interval onset of 7 years. In this scenario, hypertension would be considered the underlying cause of death because it originated before, and was considered to influence, the other causes of death. In the same example, acute myocardial infarction and atherosclerotic coronary artery disease would be considered contributing causes. Data within CDC Wonder are made available for the purposes of statistical analyses and reporting and are owned/curated by the CDC and its collaborating partners.

For the 1999–2016 data set, underlying cause of death is coded using the international classification of diseases, 10^th^ revision (ICD-10), which went into effect for mortality coding in 1999 [10]. For the purposes of this study, the primary objective was to analyze and describe trends in hypertension-related mortality between 1999–2016 in which hypertensive diseases (ICD-10 codes: essential hypertension I10, hypertensive heart disease I11, hypertensive renal disease I12, hypertensive heart and renal disease I13, secondary hypertension I15) were listed as the underlying cause of death. Please note that underlying cause of death is defined as the disease or injury that initiated the chain of events which ultimately led to death [9]. Initial analyses focused on individuals 35–85 years of age and older. Those less than 35 years of age had hypertension-related mortality rates consistently below 1/100,000 and were excluded from analysis. Due to a significant increase in mortality for those 55–85 years of age and older, as described in the results, later analyses focused on this age range and excluded those 54 years of age and younger.

Mortality rates were age-adjusted using the 2000 U.S. standard population, and were further validated using 2010 and 2016 population estimates [11]. Data on age, sex, race (Asian or Pacific Islander, Native American or Alaska Native, Black or African American, and White American) and ethnicity (Hispanic), and geographic area (census region and state) were obtained from CDC WONDER. Data from states and counties designated as Contract Health Service Delivery Areas were utilized for analysis of Native American or Alaska Native trends to mitigate racial misclassification [12].

### Trend analysis

Temporal trends in mortality were analyzed using the National Cancer Institute’s Joinpoint trend analysis software [13] (version 4.6.0.0, https://surveillance.cancer.gov/joinpoint/). The joinpoint, or segmented linear regression, model was chosen for this study because our primary goal was to delineate trends in average annual percent change (AAPC) in hypertension-related mortality rates and determine whether there were specific time intervals where the AAPC may have changed. In this regard, the joinpoint model facilitates easy interpretation as well as provides robust evidence of specific linear trends due to its grid search and permutation testing (discussed below). In addition, we were interested in modeling and interpreting trends from specific categories of predictor variables such as age, race, sex, and location, which is possible with the joinpoint model.

Briefly, the software uses a segmented linear regression model with a grid search method to analyze temporal trends in mortality rates and determines whether rate changes can be best described by a straight line (0 joinpoints) or by 1 or more linear segments (changes in slope), indicating a significant change in rate [13–15]. The software reports the simplest model that can be fit (based on minimum number of joinpoints within set limit) to explain mortality rate trends. Tests of significance (α = 0.05) for rate change are conducted using the Monte Carlo Permutation method (4499 permutations), with a Bonferroni correction for multiple testing. To ensure rate changes in mortality were due to consistent changes over time and not year-to-year variation, the minimum number of observations between two joinpoints was set to three years, and the maximum number of joinpoints within a model was set to three, which is determined by the joinpoint software. Changes in rate for a given linear segment are presented as the AAPC with 95% confidence interval.

### Underlying and contributing cause analysis

In addition to underlying cause trends, mortality trends for common contributing causes of death attributable to an underlying cause of hypertension were assessed. Death certificates have one underlying cause of death with up to 20 contributing causes. Mortality data with an underlying cause of hypertension were examined for specific contributing causes, including atrial fibrillation (I48), cardiac arrest (I46), heart failure (I50), chronic obstructive pulmonary disease (COPD unspecified, J44.9), non-insulin-dependent diabetes mellitus (E11), obesity (E66), and vascular dementia (FO1). Mortality data for each contributing factor were obtained by selecting for an underlying cause of hypertension (I10-I15), and then individually pulling data on single contributing causes.

Trend analysis for common underlying causes of death with a contributing cause of hypertension were also conducted. Underlying causes included atherosclerotic heart disease (I25.1), myocardial infarction (I21), stroke (I64), Alzheimer’s disease (G30), COPD unspecified (J44.9) [16], Parkinson’s disease (G20), non-insulin-dependent diabetes (E11), vascular dementia (F01) [17], and falls (W00-W19) [18].

### Mortality rate ratio testing

Hypertensive mortality rate ratios (MRR) for 1999–2010 and 2011–2016 were calculated for various demographic factors by dividing the number of deaths attributable to hypertension by the total number of deaths for that particular time period. Age-adjusted rates were used for all characteristics analyzed except for age-group comparisons. Mortality rate ratios were also compared for various underlying causes of death with a contributing cause of hypertension (see above). However, these analyses excluded data from 1999 due to unreliability in data for that year. This year was considered unreliable because deaths with a contributing cause of hypertension rose 115% (183.3 vs 390.8/100,000) between 1999 and 2000, which we viewed as a potential inaccuracy that could bias contributing cause analysis. In addition, analysis of contributing cause data using statistical control charts further showed that contributing cause data from 1999 had a probability of at least 1 in 1000 of resulting from a similar distribution as data from 2000–2016 [19]. This trend was not apparent in statistical analysis of underlying cause data (S1 Fig). Although we are unsure of the cause of a potential inaccuracy in contributing cause coding for hypertension in 1999, we speculate this inaccuracy may have been due to a change from ICD-9 to ICD-10 coding and a potential unfamiliarity with ICD-10 coding at that time.

Chi-squared tests with Yates correction were used to compare differences in time periods. P values of <0.05 were considered statistically significant.

### Graphing

All graphing was performed in GraphPad Prism (version 6.0) software (GraphPad Software Inc.) or Python (Python Software Foundation. Python Language Reference, version 3.7). U.S. geographical maps of mortality rates were created using the JMP Pro (version 13.2.1) statistical discovery package (SAS Institute Inc.).

### Patients

Patients were not involved at any point in this study (research question, design, analysis, interpretation of results). There are no specific plans to distribute information presented to a specific community or communities. The intention is to provide up-to-date mortality trends to aid in future decision making.

## Results

### Hypertensive mortality burden

Between 1999 and 2016, a total of 1,079,913 deaths were assigned to an underlying cause of hypertension (42,625 in 1999 and 82,057 in 2016, 92.5% increase in total deaths) (Table 1). The age-adjusted mortality rate grew 36.4% from 1999 (30.6 deaths/100,000 individuals) to 2016 (41.8/100,000) in individuals 35–85+ years of age. All age groups between 35–85+ years showed increased mortality during this period, with the largest percentage increase occurring in the age groups of 35–44 years (74.1%), 45–54 years (87.3%), and 55–64 years (68.4%). Females accounted for more cumulative deaths between 1999–2016, but males had a greater overall burden of age-adjusted mortality. Between 1999 and 2016, the age-adjusted mortality ratio between men and women grew from 1:1 to 1.24:1. When stratified by age group (S2 Fig), women 85 years and older displayed the greatest mortality rate due to hypertension (517.8/100,000 in 2016), although all age groups for both sexes exhibited increased mortality between 1999 and 2016. Regarding race, the greatest burden of age-adjusted mortality due to hypertension was noted in Black or African American individuals (83.9/100,000) followed by American Indian or Alaska Native (36/100,000), White (31.5/100,000), and Asian or Pacific Islander (26.9/100,000). Interestingly, between 1999 and 2016, there was no significant increase in age-adjusted mortality for the Black or African American population (0.1%), while there were increases in both the American Indian or Alaska Native (167%) and White (46.5%) populations, although the White demographic accounted for significantly more total deaths compared to the American Indian or Alaska Native group. In addition, the age-adjusted hypertension-related mortality rate was greater than 30 for both Hispanic (32.2/100,000) and Non-Hispanic (36.6/100,000) ethnicity. From 1999–2016, there was an 86.5% and 26.2% increase in mortality for the Non-Hispanic and Hispanic demographics, respectively. Geographically, all census regions (Northeast, Midwest, South, and West) were observed to have age-adjusted mortality rates greater than 30, with the greatest occurring in the South (40.1/100,000) and West (38/100,000). Moreover, all census regions showed similar increases in age-adjusted mortality between 1999 and 2016.

**Table 1:** Trends in hypertension mortality 1999–2016 for individuals 35 years of age and older.

Characteristics	Deaths	Age-adjusted Rate* (95% CI)	Deaths	Age-adjusted Rate* (95% CI)		Deaths	Age-adjusted Rate* (95% CI)	Death rate percent change (1999–2016)
Year	Cumulative (1999–2016)		1999			2016		
**Overall**	1079913	36.39 (36.31–36.47)	42625		30.67 (30.38–30.96)	82057	41.84 (41.55–42.13)	36.40%
**Age Group:**								
35–44 years	33029	4.32 (4.27–4.36)	1391		3.09 (2.92–3.25)	2177	5.38 (5.15–5.61)	74.10%
45–54 years	89911	11.79 (11.72–11.87)	2885		7.89 (7.6–8.18)	6322	14.78 (14.41–15.14)	87.30%
55–64 years	136228	22.81 (22.69–22.93)	4117		17.31 (16.79–17.84)	12091	29.16 (28.64–29.68)	68.40%
65–74 years	155818	40.34 (40.14–40.54)	6786		36.84 (35.97–37.72)	13706	47.87 (47.07–48.67)	29.90%
75–84 years	251540	106.59 (106.18–107.01)	12241		100.13 (98.36–101.91)	16550	116.27 (114.50–118.05)	16.10%
≥ 85 years	413387	443.37 (442.01–444.72)	15205		366.03 (360.21–371.85)	31211	489.18 (483.75–494.60)	33.60%
**Sex:**								
Female	605415	33.58 (33.50–33.67)	25686		29.55 (29.19–29.92)	42955	37.11 (36.75–37.47)	25.50%
Male	474498	38.45 (38.33038.56)	16939		30.72 (30.25–31.19)	39102	46.32 (45.85–46.78)	50.70%
**Race:**								
American Indian or Alaska Native	3664	36.02 (34.78–37.27)	72		19.18 (14.81–24.45)	403	51.22 (45.97–56.48)	167.00%
Asian or Pacific Islander	25765	26.95 (26.61–27.28)	802		27.54 (25.54–29.54)	2549	29.17 (28.03–30.32)	5.90%
Black or African American	231504	83.93 (83.58–84.28)	10165		83.47 (81.82–85.11)	16322	83.57 (82.25–84.89)	0.10%
White	817499	31.53 (31.46–31.6)	31535		25.48 (25.2–25.76)	62650	37.35 (37.05–37.64)	46.50%
Hispanic	63314	32.27 (32.01–32.54)	1795		27.38 (26.05–28.71)	5832	34.57 (33.65–35.49)	26.20%
Non-Hispanic	1012413	36.64 (36.57–36.71)	40632		30.70 (30.41–31.00)	75818	42.56 (42.25–42.87)	86.50%
**Census Region**								
Northeast	185660	30.83 (30.69–30.97)	7672		26.02 (25.43–26.6)	13569	35.93 (35.32–36.55)	38.00%
Midwest	232633	33.91 (33.77–34.05)	9646		28.73 (28.16–29.31)	16920	39.27 (38.66–39.87)	36.60%
South	423943	40.16 (40.04–40.28)	16548		34.1 (33.58–34.62)	33144	46.01 (45.51–46.51)	34.90%
West	237677	38.05 (37.9–38.21)	8759		32.07 (31.4–32.74)	18424	42.22 (41.6–42.84)	31.60%

*Age-adjusted mortality rates were calculated using the 2000 standard population for individuals 35–85+ years of age.

### Trends in hypertensive mortality

**Fig 1:**
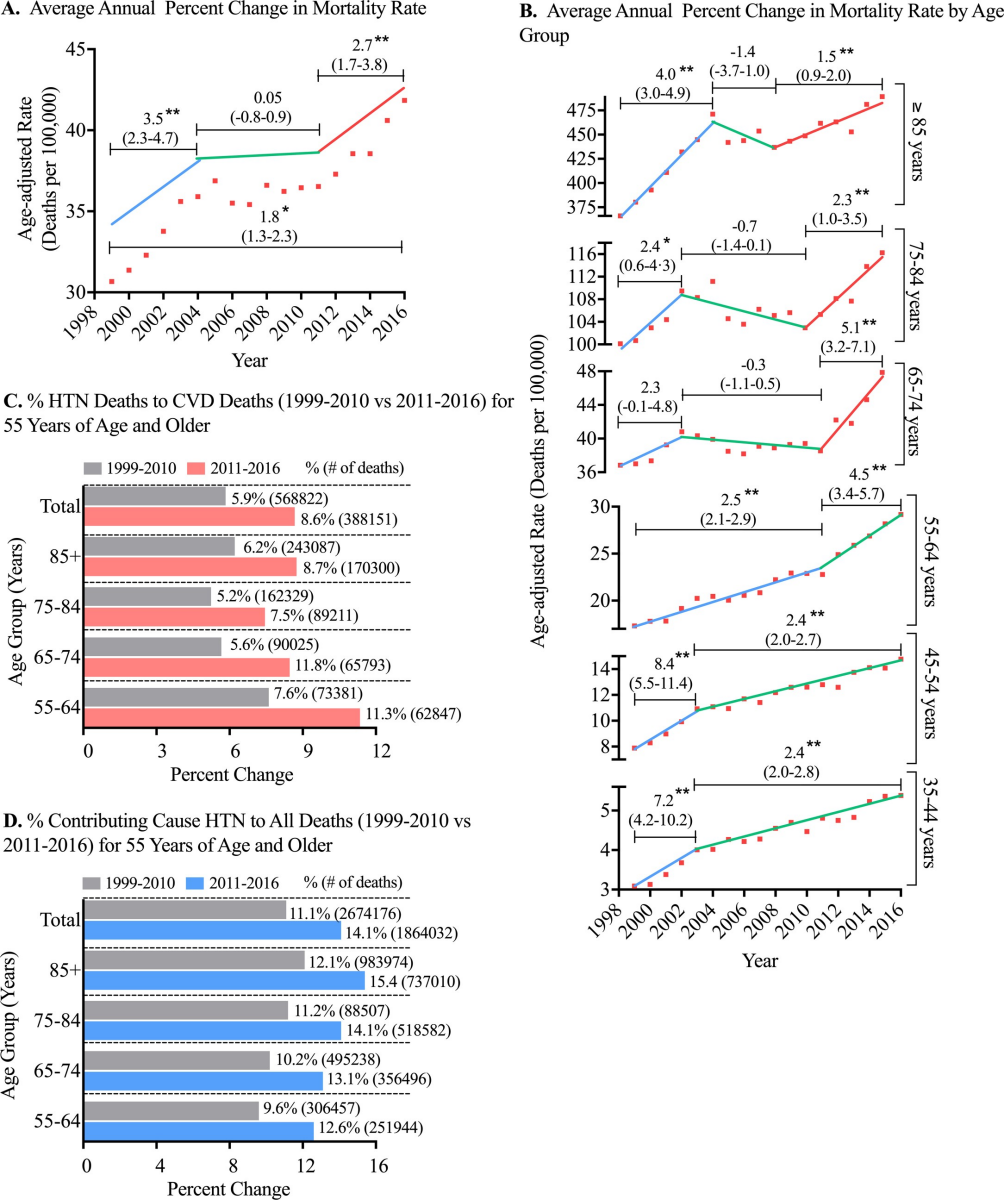
Annual trends in mortality due to hypertension. (**A**) Analysis of average annual percent change for hypertension-related mortality 1999–2016. Analysis was conducted using joinpoint regression. (**B**) Analysis of mortality data from 1999–2016 stratified by 10-year age group. (**C**) Deaths attributable to hypertension as a percentage of total cardiovascular deaths (1999–2010 vs 2011–2016). Cardiovascular deaths were defined as disease of the circulatory system (ICD10: I00-I99). (**D**) Deaths with a contributing cause of hypertension as a percentage of all deaths. Deaths with an underlying cause of hypertension were excluded from analysis for (**D**) to avoid counting deaths with both underlying/contributing hypertension. Results for **A** and **B** are presented as mean AAPC±95% confidence interval. *p<0.05,**p<0.01.

To further evaluate the burden of hypertensive mortality over time, we analyzed trends in mortality rate changes from 1999–2016 in individuals 35–85+ years of age. Between 1999 and 2016, there were 3 notable trends in the average annual percent change (AAPC) of age-adjusted mortality rates: (1) a statistically significant increase of 3.5±1.2% (mean±95% confidence interval) per year between 1999 and 2004, (2) a leveling off between 2004 and 2011, (3) and a 2.7±1.1% increase per year between 2011 and 2016 (Fig 1A). These trends held constant when mortality rates were standardized to both 2010 and 2016 population estimates (S3 Fig).

We next sought to assess the contribution of age to the increase in the AAPC of hypertensive mortality between 2011–2016. Interestingly, from 2003–2016, individuals 54 years of age and younger displayed a reduced AAPC, whereas individuals 55 years and older all exhibited increased AAPC between 2011 and 2016 (Fig 1B). The greatest change was noted in those 55–64 (AAPC 4.5±1.1%), and 65–74 (AAPC 5.1±1.9%) years of age. These data suggest the uptick in overall mortality between 2011–2016 may be attributable to individuals 55 years and older. This observation is especially concerning considering the 65+ age groups showed plateaus in mortality rates prior to that inflection. In line with these findings, the percent contribution of hypertension-related deaths (listed as underlying) to cardiovascular-related deaths rose from 5.9% between 1999 and 2010 to 8.6% between 2011 and 2016 for the 55–85+ age group (Fig 1C). Likewise, the percent contribution of deaths with a contributing cause of hypertension to all deaths increased from 11.1% to 14.1% for the same time period and age group (Fig 1D).

Summarizing the data presented in Table 1 and Fig 1, there was a significant increase in hypertension-related mortality from 1999–2016, and from 2011–2016 there was an acceleration in the AAPC of hypertension-related mortality for those 55 years of age and older, which accounted for the largest population and greatest number of deaths in the study period. Given these findings, we further explored trends in the 55 and older age group and the 2011–2016 time period.

### Demographic-related changes in hypertensive mortality for the 55 and older age group

**Fig 2:**
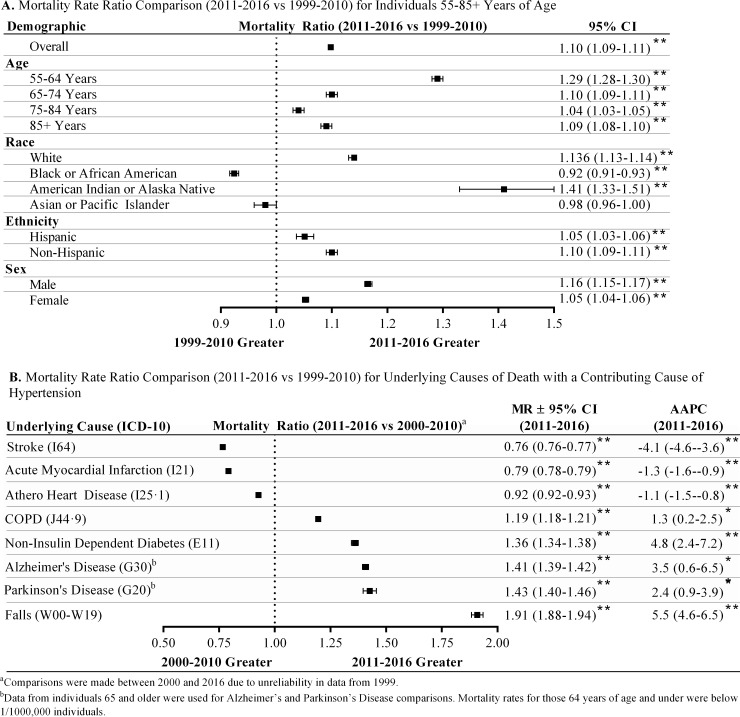
Demographic and disease trends for hypertension-related mortality. (**A**) Mortality rate ratios using hypertension as an underlying cause of death from 2011–2016 and 1999–2010 were calculated for various demographic factors and compared. (**B**) Mortality rate ratios were calculated for common underlying causes of death with a contributing cause of hypertension and were compared. Joinpoint regression analysis was also performed to delineate changes in mortality rate for 2011–2016. Chi-square tests were used to analyze significant differences between mortality rate ratios. All results are presented as mean±95% confidence interval. *p<0.05, **p<0.01.

Underlying cause hypertensive mortality rates were further subdivided by age, race, sex, and ethnicity for the 2011–2016 time period and 55 and older age group in order to gain insight into demographic trends. Initial comparison of mortality rate ratios between 1999–2010 and 2011–2016 showed significant increases in all demographic groups except for Black or African American (8% decrease) and Asian or Pacific Islander race (Fig 2A). The greatest increases in mortality rate ratios were observed for those 55–64 years of age (MRR 1.29±0.01), those of White (MRR 1.13±0.01) or American Indian or Alaska Native (MRR 1.41±0.1) race, non-Hispanic ethnicity (MRR 1.10±0.01), and male sex (MRR 1.16±0.01). In addition, increased AAPC in hypertensive-related mortality was noted in both male (AAPC 3.5±1%) and female (AAPC 2.0±1.1%) sex, American Indian or Alaska Native (AAPC 7.3±3.1%) and White (AAPC 2.9±1.1%) race, and those of non-Hispanic ethnicity (AAPC 2.7±1.0%) (Table 2). Increased AAPC in these groups was also pervasive throughout ten-year age groupings. Thus, between 2011 and 2016, there were significant increases in both the overall rate of hypertension-related mortality and its AAPC across all demographics except for Black or African American and Asian or Pacific Islander races.

**Table 2:** Trends in AAPC for underlying hypertension mortality for individuals 55–85+ years of age (2011–2016).

	Overall	55–64	65–74	75–84	85+
**Overall**	2.7 (1.7–3.7)**	4.7 (3.6–5.9)**	4.2 (2.1–6.3)**	2.4 (1.6–3.3)**	1.6 (0.2–2.9)*
**Sex**					
Female	2.0 (0.9–3.2)**	4.6 (2.0–6.1)**	3.3 (0.7–6.0)*	1.8 (0.8–2.8)**	1.2 (-0.2–2.7)
Male	3.5 (2.5–4.5)**	4.8 (3.4–6.2)**	4.8 (2.9–6.7)**	3.2 (2.2–4.2)**	2.6 (1.0–4.1)*
**Race**					
American Indian or Alaska Native	7.3 (4.2–10.4)**	12.5 (-2.1–29.3)	7.7 (2.9–12.7)*	11.3 (4.2–18.9)*	1.9 (-2.8–6.9)
Asian or Pacific Islander	1.6 (-1.9–5.1)	6.8 (2.9–10.8)*	1.9 (-0.3–4.1)	1.9 (-2.6–6.6)	0.7 (-3.8–5.3)
Black or African American	0.9 (-0.2–1.9)	1.7 (0.4–3.0)*	1.2 (-1.1–3.6)	1.8 (0.1–3.5)*	-0.8 (-2.8–1.2)
White	2.9 (1.9–4.0)**	5.7 (4.4–7.0)**	5.2 (3.1–7.3)**	2.4 (1.5–3.3)**	1.9 (0.6–3.1)*
Hispanic	1.6 (-0.3–3.6)	3.7 (-0.4–7.8)	3.0 (0.4–5.5)*	1.2 (-0.9–3.2)	1.0 (-1.6–3.7)
Non-Hispanic	2.7 (1.7–3.7)**	4.9 (3.9–5.9)**	4.3 (2.1–6.5)**	2.5 (1.6–3.4)**	1.6 (0.2–3.0)*

*p<0.05

**p<0.01.

Age-adjusted mortality rates were calculated using the 2000 standard population for individuals 55–85+ years of age.

### Trends in contributing causes of death and hypertension

Hypertension is associated with varying comorbidities, and so common contributing causes of death with an underlying cause of hypertension were examined for the 55 and older age group. Between 2011 and 2016, there were significant increases in the AAPC for hypertension-related mortality attributable to contributing causes of atrial fibrillation (AAPC 6.7±1.1%), cardiac arrest (AAPC 2.5±1.2%), heart failure (AAPC 4.2±1.5%), COPD (AAPC 4.9±2.1%), non-insulin dependent diabetes mellitus (AAPC 8.0±3.6%), and obesity (AAPC 8.7±2.3%) (Table 3). A significant increase in a contributing cause of vascular dementia was also observed, although this trend was only significant for the 85+ age group. Further analysis on common contributing causes by age group is presented in Table 3 and S4–S7 Figs.

**Table 3:** Trends in contributing causes with an underlying cause of hypertension (2011–2016).

Contributing Cause (ICD 10)	Overall	55–64	65–74	75–84	85+
**Atrial Fibrillation (l48)**					
Mortality Rate	13.72 (13.59–13.86)	0.69 (0.66–0.73)	2.41 (2.33–2.49)	11.67 (11.43–11.90)	67.75 (66.91–68.60)
AAPC	6.7 (5.6–7.8)**	NA	8.4 (4.8–12.2)**	7.0 (5.3–8.8)**	6.4 (5.5–7.3)**
**Cardiac Arrest (l46)**					
Mortality Rate	18.39 (18.27–18.51)	4.95 (4.86–5.04)	10.00 (9.84–10.15)	26.65 (26.30–27.01)	105.98 (104.93–107.04)
AAPC	2.5 (1.3–3.7)**	4.6 (3.4–5.8)**	3.2 (0.3–6.2)*	2.2 (1.3–3.1)**	1.9 (0.1–38)*
**Heart Failure (l50)**					
Mortality Rate	16.83 (16.71–16.95)	1.87 (1.82–1.93)	5.19 (5.07–5.30)	21.94 (21.62–22.26)	135.72 (134.52–136.92)
AAPC	4.2 (2.7–5.7)**	7.3 (4.4–10.4)**	5.4 (3.4–7.4)**	3.9 (2.4–5.4)**	4.0 (2.2–5.7)**
**COPD (J44.9)**					
Mortality Rate	6.04 (5.97–6.11)	2.17 (2.12–2.23)	4.29 (4.18–4.39)	9.61 (9.40–9.83)	24.85 (24.34–25.36)
AAPC	4.9 (2.8–7.1)**	8.1 (4.6–11.6)**	6.2 (1.9–10.7)*	4.1 (1.3–6.9)*	3.3 (0.7–6.0)*
**Non-Insulin-Dependent Diabetes Mellitus (E11)**					
Mortality Rate	2.47 (2.43–2.52)	0.68 (0.64–0.71)	1.55 (1.49–1.61)	3.97 (3.83–4.11)	12.16 (11.80–12.52)
AAPC	8.0 (4.4–11.7)**	NA	9.4 (2.7–16.5)*	8.4 (3.8–13.2)#x002A;*	6.3 (4.4–8.2)**
**Obesity (E66)**					
Mortality Rate	2.20 (2.16–2.63)	2.55 (2.49–2.62)	2.21 (2.13–2.28)	1.66 (1.58–1.75)	1.42 (1.30–1.54)
AAPC	8.7 (6.4–11.1)**	6.8 (3.0–10.7)**	11.0 (7.9–14.3)**	11.7 (4.7–19.2)**	6.5 (3.6–9.5)**
**Vascular Dementia (F01)**					
Mortality Rate	1.39 (1.36–1.42)	0.03 (0.02–0.04)	0.24 (0.21–0.26)	1.86 (1.77–1.95)	12.81 (12.44–13.17)
AAPC	4.1 (-0.5–8.8)	NA	NA	1.8 (-5.3–9.5)	4.8 (0.8–9.0)*

*p<0.05

**p<0.01.

Age-adjusted mortality rates were calculated using the 2000 standard population.

For AAPCs labeled “NA”, AAPC was not conducted due to too few deaths (less than 1/100,000) or an absence of a CDC reported rate.

We also examined any underlying cause of death with a contributing cause of hypertension, which accounted for ~ 6 million deaths from 2000–2016 (S8A Fig). Although the listing of a contributing cause of death is prone to variability in accuracy, a trend of increased mortality was noted for individuals 55–74 years of age between 2009–2016 (S8B Fig) that was similar to our observation of increased rates of mortality for deaths with a underlying cause of hypertension for these age groups. Interestingly, a decline in mortality associated with a contributing cause of hypertension was noted for individuals 75 and over after 2009, which is in contrast with our earlier findings (Fig 1). Similar to the analysis presented in Fig 2A, mortality rate ratios for common underlying causes of death associated with a contributing cause of hypertension were examined for those 55 years and older. Compared to 2000–2010 (data from 1999 were determined unreliable), common underlying causes of death including stroke, acute myocardial infarction, and atherosclerotic heart disease all showed reduced mortality rate ratios and negative AAPCs between 2011–2016, indicating a reduction in overall mortality and continually declining mortality rates (Fig 2B). However, significant increases in mortality rate ratios and AAPCs were noted for COPD (MRR 1.19±0.01, AAPC 1.3±1.1%), non-insulin dependent diabetes (MRR 1.36±0.02, AAPC 4.8±2.4%), Alzheimer’s Disease (MRR 1.41±0.01, AAPC 3.5±3.0%), Parkinson’s Disease (MRR 1.43±0.03, AAPC 2.4±1.5%), and falls (MRR 1.91±0.03, AAPC 5.5±1.0%) from 2011–2016. Mortality rates for each age-group by year are presented in S9–S11 Figs. The increase in fall-related mortality with a contributing cause of hypertension is particularly concerning considering the reported risk of falling or balance impairment in various populations of hypertensive individuals, especially those receiving antihypertensive medication [18, 20, 21]. Overall, these data suggest an increasing association between mortality due to hypertension and common contributing causes of death, as well as an increased association between a contributing cause of hypertension and underlying causes of death including neurological and metabolic disorders, and balance impairment.

### Geographic and state trends in hypertensive mortality

**Fig 3:**
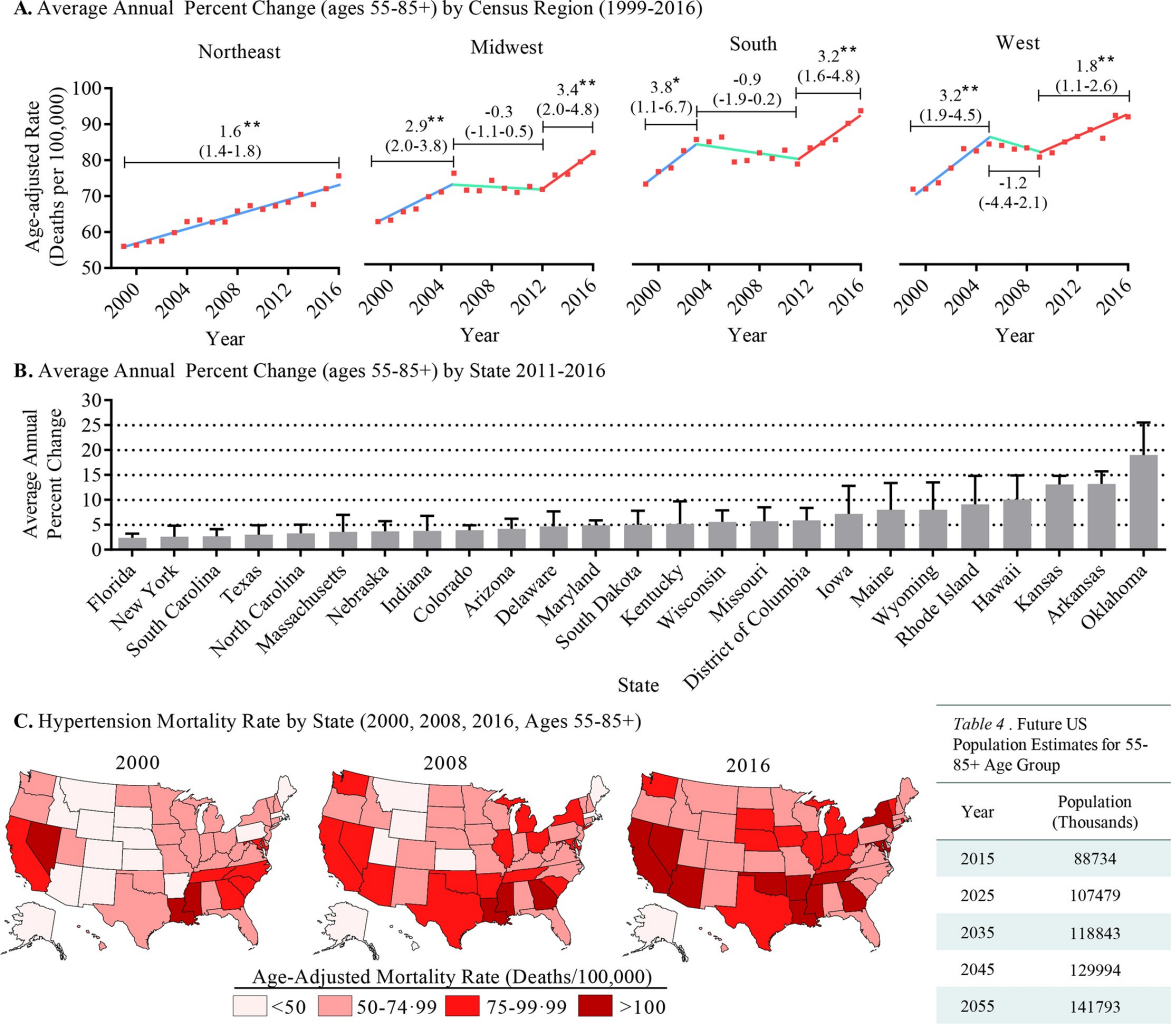
Geographic trends in hypertension-related mortality. (**A**) Mortality trends by census region for the 55–85+ age group between 1999 and 2016. (**B**) U.S. State-specific changes in AAPC between 2011 and 2016 for the 55–85+ age group. (**C**) U.S. Heatmaps for the years 2000, 2008, and 2016 displaying age-adjusted mortality rates for the 55–85+ age group. The table shows population estimates, in 10-year intervals, for the 55–85+ age group. Estimates were acquired from the U.S. Census Bureau. Results, including bar graphs, are presented as mean AAPC±95% confidence interval. *p<0.05, **p<0.01.

Hypertension mortality data from the 55 and older age group were also analyzed for geographical trends. All census regions showed statistically significant increases in AAPC between 1999 and 2016; however, the Midwest, South, and West all experienced plateaus in the mid 2000’s followed by significant inflections in AAPC starting in the late 2000’s (2009–2012), which remained through 2016 (Fig 3A). In addition, between 2011 and 2016, 25 out of 51 states (Washington DC is included as a state in CDC WONDER database) experienced significant increases in AAPC of hypertension-related mortality (Fig 3B); all of which had mortality rates greater than 40/100,000, and 13 of which had an AAPC of 5% or greater (S12 Fig). Using State heatmaps, changes in hypertension-related mortality rates for all states since 2000 are evident (Fig 3C). By 2016, 50/51 states had a mortality rate above 50/100,000, 23 states had a mortality rate of 75/100,000 or above, and 12 states had a mortality rate above 100/100,000. This trend is striking compared to 2000 and 2008, where only 4 states were reported to have a mortality rate above 100/100,000 for the 55 and older age group. These data indicate a trend of growing state AAPC and hypertension-related mortality rates for individuals 55 years of age and older.

## Conclusions

Hypertension with its associated complications is a severe and chronic condition that contributes to a high percentage of deaths [22]. The data presented are in line with previous reports indicating an overall increase in hypertension-related mortality for previous time periods [1, 6, 23, 24]; however, our data add important missing information that extends the breadth of existing literature. The following discussion should also be taken in the context that causation of death was established based on documentation of underlying cause of death on death certificates, which may be prone to bias and unreliability.

First, our analysis is the first to examine the rate of change of hypertension mortality over the entire 1999 to 2016 time period, and we find that the AAPC has been increasing in recent years, suggesting possibly worsening outcomes for individuals with hypertension. Importantly, we find that the increase in AAPC from 2011–2016 is primarily attributable to the 55 and older age group, and that this group experienced a 91.8% increase in annual hypertension-related mortality between 1999 and 2016 and a 29.4% increase from 2011–2016. The significant increase in mortality is alarming considering the population size of the 55 and older age group is estimated to increase over the next 30 years (Fig 3C). An increasing mortality rate, with an accelerating rate of change, coupled with an increasing population size for this group may have severe economic and healthcare ramifications in the United States that far exceed current healthcare resource and expenditure estimates [25].

Second, our data provide comprehensive and granular insight into demographic and geographic trends in hypertension-related mortality. In particular, we observe numerous demographic groups to have increased AAPCs from 2011–2016. Aside from increases in the 55 and older age group, increased mortality rates and AAPC were noted for both male and female sex, White and American Indian or Alaska Native race and non-Hispanic ethnicity. Our data are also in line with previous findings indicating reduced mortality rates for those of Black or African American race [1], and our data extend these findings by showing a plateau in AAPC between 2011 and 2016 for these individuals. However, it should be noted that although a significant trend for Black or African Americans was not found in our analysis, this group did experience an increase in overall death counts during the 2011–2016 period. High year-to-year variation and a high mortality rate (small changes in counts have a small effect on the overall rate) are potential reasons for a lack of significant increase. Another possible explanation for this plateau may be due to an increase in blood pressure control and treatment for this group, although further analysis is needed [26].

Our data also point to a significant increase in state hypertension-related mortality rates and AAPCs during the study period. From 2011–2016, 25 states were found to have increased AAPCs, and in 2016, 50/51 states had a mortality rate above 50/100,000. The reported findings add to preexisting information concerning hypertension prevalence by demographic grouping and geographic location by providing new and updated data on mortality trends within the United States. The widespread increase in mortality across all states poses a significant challenge to national care, policy making and implementation, and resource allocation. Specific decisions must account for both geographic and national trends.

Lastly, our analyses point towards increases in association of some common causes of death with hypertension. Importantly, our data indicate decreases in mortality due to stroke, myocardial infarction and heart disease with a contributing cause of hypertension, and increases in mortality due to COPD, non-insulin dependent diabetes, Alzheimer’s, Parkinson’s, and falls with a contributing cause of hypertension. The continual assessment of trends in disease association could help identify contributing factors to disease progression and mortality, which may have not been previously evident. Together, the presented findings provide valuable information that could aid in the assessment of how well hypertension is being managed across the United States, as well as identify areas of immediate and future action.

It is important to interpret these data within the limitations of the research analysis. The reported findings are based on data acquired from death certificates (reported in CDC WONDER), which have varying degrees of inaccuracy and bias, depending upon type of disease [27, 28]. In the United States, the accuracy of hypertension diagnosis as an underlying cause of death is currently unknown. However, even with unknown accuracy, the large sample size used in this study should be sufficient to depict overall secular trends if the associated error is random. This assumption does not hold true if error is due to bias, which is unknown and warrants further investigation. There is also the possibility that our findings are a result of aggregation bias, as we utilized 10-year age groups [29]. However, AAPC analysis of single year age groups from 1999 to 2016 showed similar increases in acceleration for various ages (S13 Fig). In addition, this study does not address why mortality may have increased in recent years, which merits further investigation. Potential explanations may include changes in prevalence, screening methods (blood pressure guidelines changed in 2003 and global) [1], a greater appreciation for the role hypertension plays in facilitating life-threatening comorbidities (physicians may be more likely to indicate hypertension as a underlying cause of death), changes in accuracy of ICD coding over time, antihypertensive medication use, blood pressure control [30], and increased sodium intake [31, 32]. Importantly, recent NHANES data have pointed to an overall increase in the burden of hypertension, although control of hypertension has improved [1, 30]. This is an interesting contradiction, in that non-Hispanic whites are associated with improved blood pressure control, but our findings indicate an acceleration in mortality for these individuals.

In addition, the use of different revisions of ICD coding may have influenced the reported data; however, ICD-10 coding for mortality was adopted by the Vital Statistics Cooperative Program in 1999. Thus, while this change could potentially affect data from the early 2000’s, it should have minimal influence on the trend analyses reported for 2011–2016. Likewise, the age-group under study may be significantly prone to economic fluctuations such as the 2008 financial crisis, which may have affected health care coverage and contributed to increased blood pressure in the following years [33], although its impact on hypertension-related mortality is unknown. Regardless of contributing factors, the consistently increasing rates of mortality highlight a need for further inquiry and planning of future healthcare initiatives.

## Perspectives

Hypertension is a key player in the development of cardiovascular disease and is increasingly associated with various causes of death. By quantifying AAPC and investigating various trends, our findings highlight that hypertension-related mortality has been accelerating in recent years for individuals 55 years of age and older. In addition, these trends were constant throughout various demographic and geographical stratifications, which emphasizes a growing national problem in need of further inquiry and preventative action.

## Supporting information

S1 FigYear-to-Year Variation in Underlying and Contributing Cause of Death.Statistical control charts of year-to-year percent change in deaths with hypertension listed as an underlying (**A**) or (**B**) contributing cause of death. The percent change for the period of 1999–2000 for **B** was found to lie outside of the Upper Control Limits (UCL) indicating this data point to be an outlier and unlikely to have come from the distribution of the remaining points. UCL and lower control limits (LCL) were calculated as:
$$\bar{X}=\frac{sum\;of\;year-to-year\;change}{n}$$(1)
$$\bar{MR}=\frac{\sum_{i=2}^{n}\left|x_{i}-x_{i-1}\right|}{n-1}$$(2)
$$\mathrm{UCL}=\bar{X}+3\frac{\bar{MR}}{1.128}$$(3)
$$\mathrm{LCL}=\bar{X}-3\frac{\bar{MR}}{1.128}$$(4)
*for UCL and LCL, *σ is estimated using moving range (MR) and the statistical control constant d*_*2*_, *where d*_*2*_ *= 1*.*128*.(JPG)

S2 Fig Gender and Age-Related Trends in Hypertensive Mortality. (JPG)

S3 Fig Hypertension Mortality Trends and 2000, 2010, and 2016 Population Standardization. Joinpoint analyses were conducted on mortality data with a underlying cause of hypertension and differing population standards. Analyses were conducted with 2000, 2010, and 2016 population standards.
(PNG)

S4 Fig Top 15 Contributing Causes of Death with Underlying Cause of Hypertension for 55–64 Years of Age (2011–2016). (PNG)

S5 Fig Top 15 Contributing Causes of Death with Underlying Cause of Hypertension for 65–74 Years of Age (2011–2016). (PNG)

S6 Fig Top 15 Contributing Causes of Death with Underlying Cause of Hypertension for 75–84 Years of Age (2011–2016). (PNG)

S7 Fig Top 25 Contributing Causes of Death with Underlying Cause of Hypertension for 85+ Years of Age (2011–2016). (PNG)

S8 Fig All Cause of Death with Contributing Cause of Hypertension (2000–2016). Mortality data were collected on all underlying causes with a contributing cause of hypertension from 2000–2016. (**A**). Data were then stratified by age group (55–64, 65–74, 75–84, and 85+) and joinpoint analyses were performed (**B**). Although all ages groups exhibited significant increases over the entire 2000–2016 range, individuals aged 55–74 exhibited increases in their rate starting in the late 2000’s.
(PNG)

S9 Fig Underlying Cause with Contributing Cause of Hypertension (1–3). Mortality data were collected on all underlying causes with a contributing cause of hypertension and stratified by age. Data were collected on atherosclerotic heart disease, myocardial infarction, and stroke. ICD-10 codes are listed in parentheses next to disease name. 1999 was excluded from analysis due to un-reliable mortality rates for a few diseases.
(JPG)

S10 Fig Underlying Cause with Contributing Cause of Hypertension (4–6). Mortality data were collected on all underlying causes with a contributing cause of hypertension and stratified by age. Data were collected on Alzheimer’s disease, COPD unspecified and Parkinson’s disease. ICD-10 codes are listed in parentheses next to disease name. 1999 was excluded from analysis due to un-reliable mortality rates for a few diseases.
(JPG)

S11 Fig Underlying Cause with Contributing Cause of Hypertension (7–9). Mortality data were collected on all underlying causes with a contributing cause of hypertension and stratified by age. Data were collected on non-insulin-dependent diabetes, vascular dementia, and falls. ICD-10 codes are listed in parentheses next to disease name. 1999 was excluded from analysis due to un-reliable mortality rates for a few diseases.
(JPG)

S12 Fig State AAPC and Mortality Rates for Individuals 55–85+ Years of Age (2011–2016). Statistically significant State AAPC for 2011–2018 was plotted with cumulative State age-adjusted hypertension-related mortality data for the years 2011–2016 for individuals 55 years of age and above.
(PNG)

S13 Fig Hypertension-related Mortality by Single Year Age Groups. (JPG)

S1 Supplemental Search Term File A word doc listing all of the search terms that were used in the CDC WONDER database (wonder.cdc.gov) for the study. (DOCX)
