# Supplementary figures and images for: An acceleration in hypertension-related mortality for middle-aged and older Americans, 1999-2016: An observational study
Source: PLoS One. 2020 Jan 15;15(1):e0225207. doi: 10.1371/journal.pone.0225207 (PMC6961854; doi:10.1371/journal.pone.0225207)

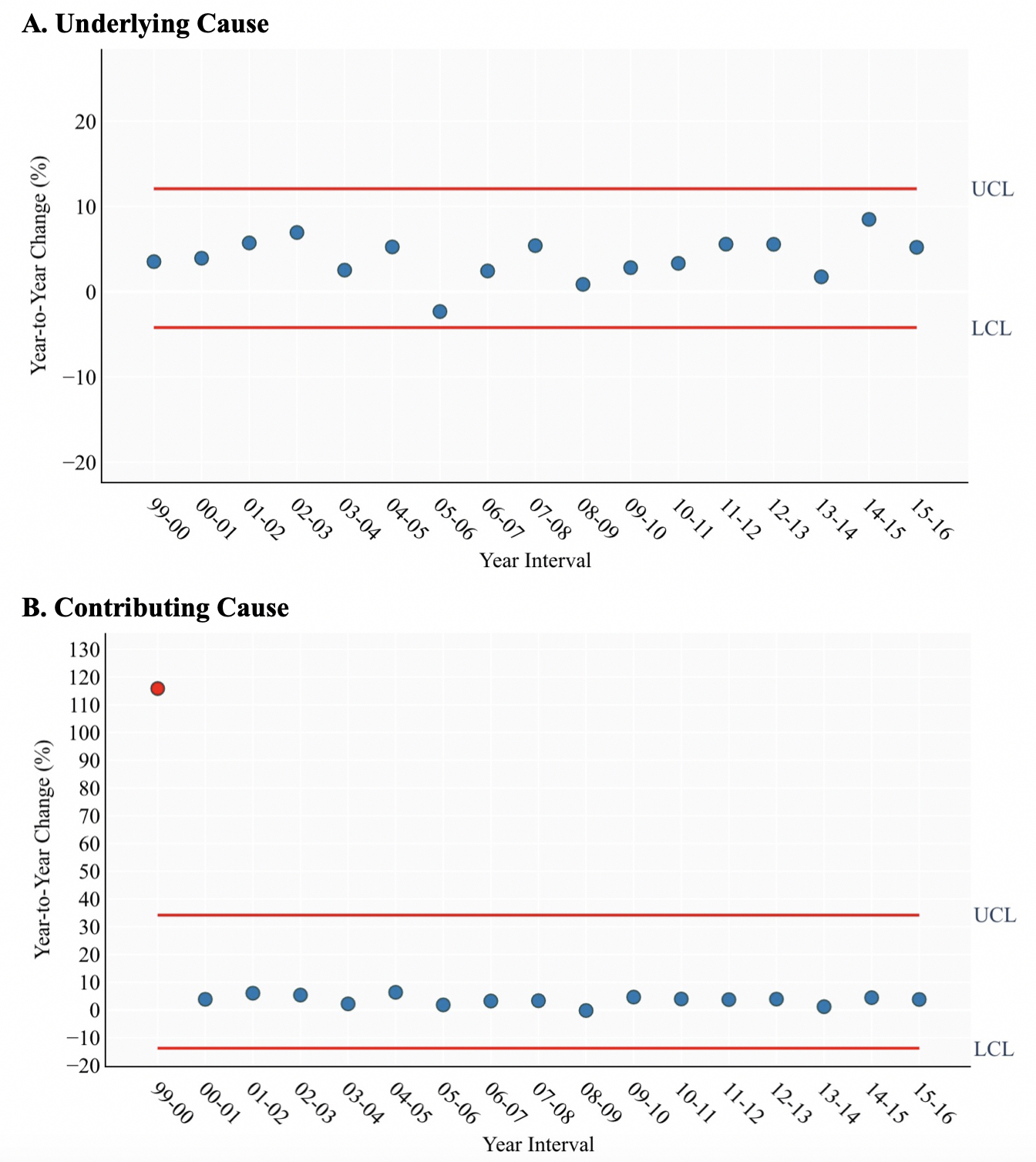

Supplement: S1 Fig — Statistical control charts of year-to-year percent change in deaths with hypertension listed as an underlying (A) or (B) contributing cause of death. The percent change for the period of 1999–2000 for B was found to lie outside of the Upper Control Limits (UCL) indicating this data point to be an outlier and unlikely to have come from the distribution of the remaining points. UCL and lower control limits (LCL) were calculated as: X¯=sumofyear−to−yearchangen(1) MR¯=∑i=2n|xi−xi−1|n−1(2) UCL=X¯+3MR¯1.128(3) LCL=X¯−3MR¯1.128(4) *for UCL and LCL, σ is estimated using moving range (MR) and the statistical control constant d2, where d2 = 1.128. (JPG) [file pone.0225207.s001.jpg]

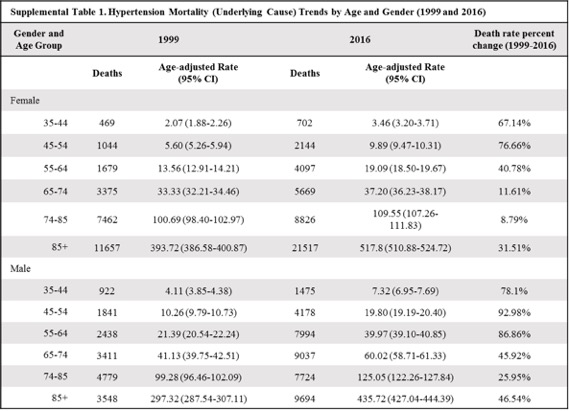

Supplement: S2 Fig — (JPG) [file pone.0225207.s002.jpg]

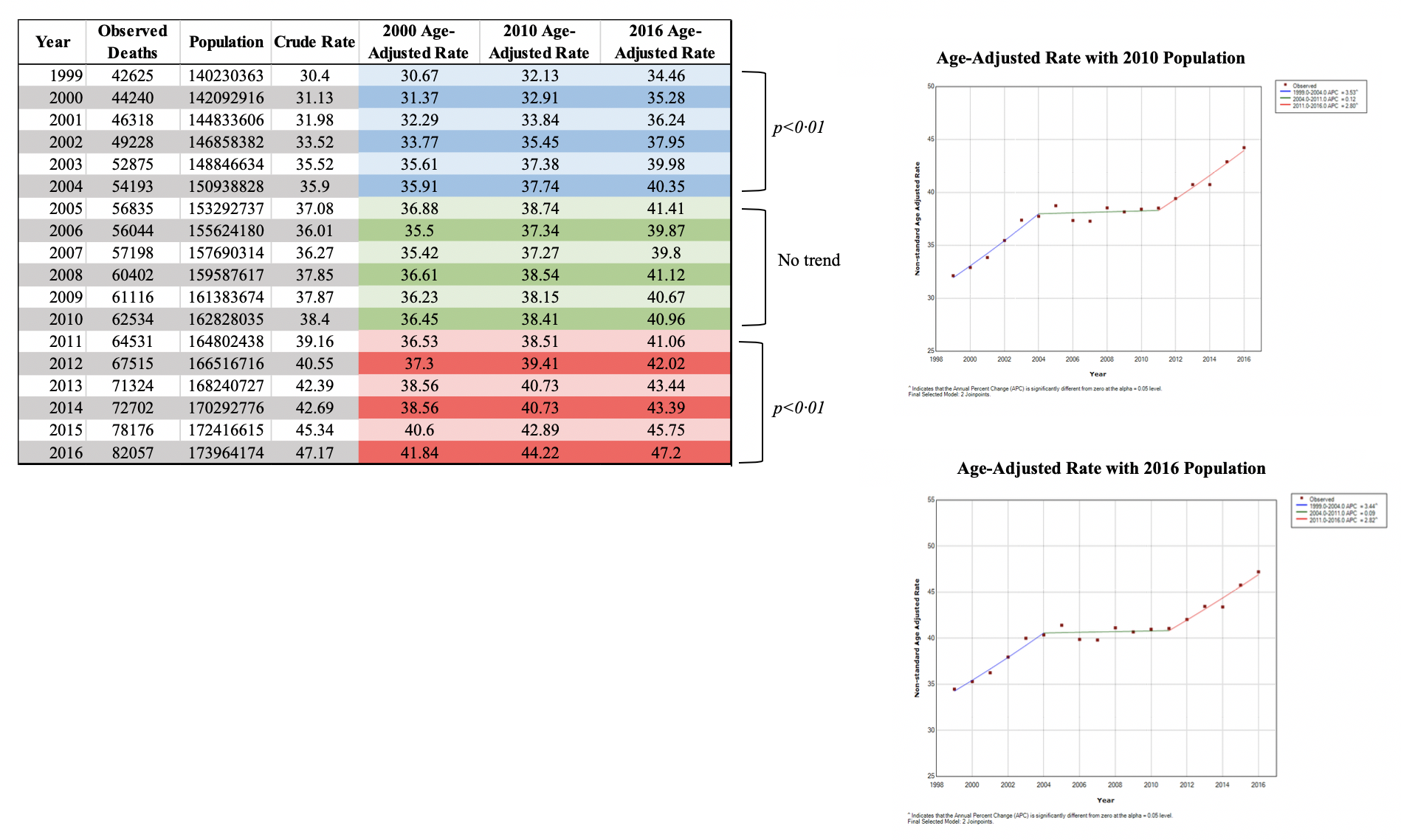

Supplement: S3 Fig — Joinpoint analyses were conducted on mortality data with a underlying cause of hypertension and differing population standards. Analyses were conducted with 2000, 2010, and 2016 population standards. (PNG) [file pone.0225207.s003.png]

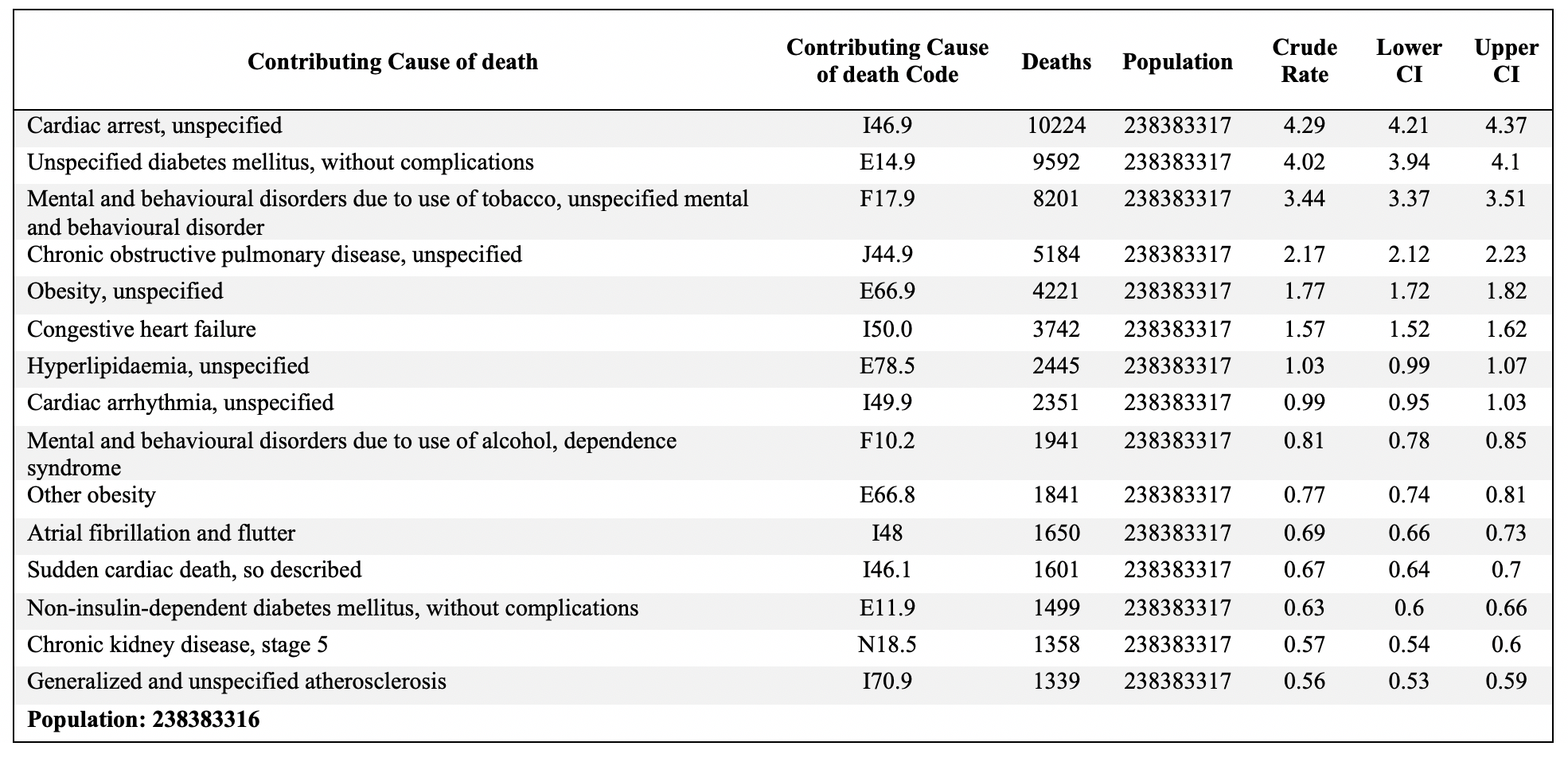

Supplement: S4 Fig — (PNG) [file pone.0225207.s004.png]

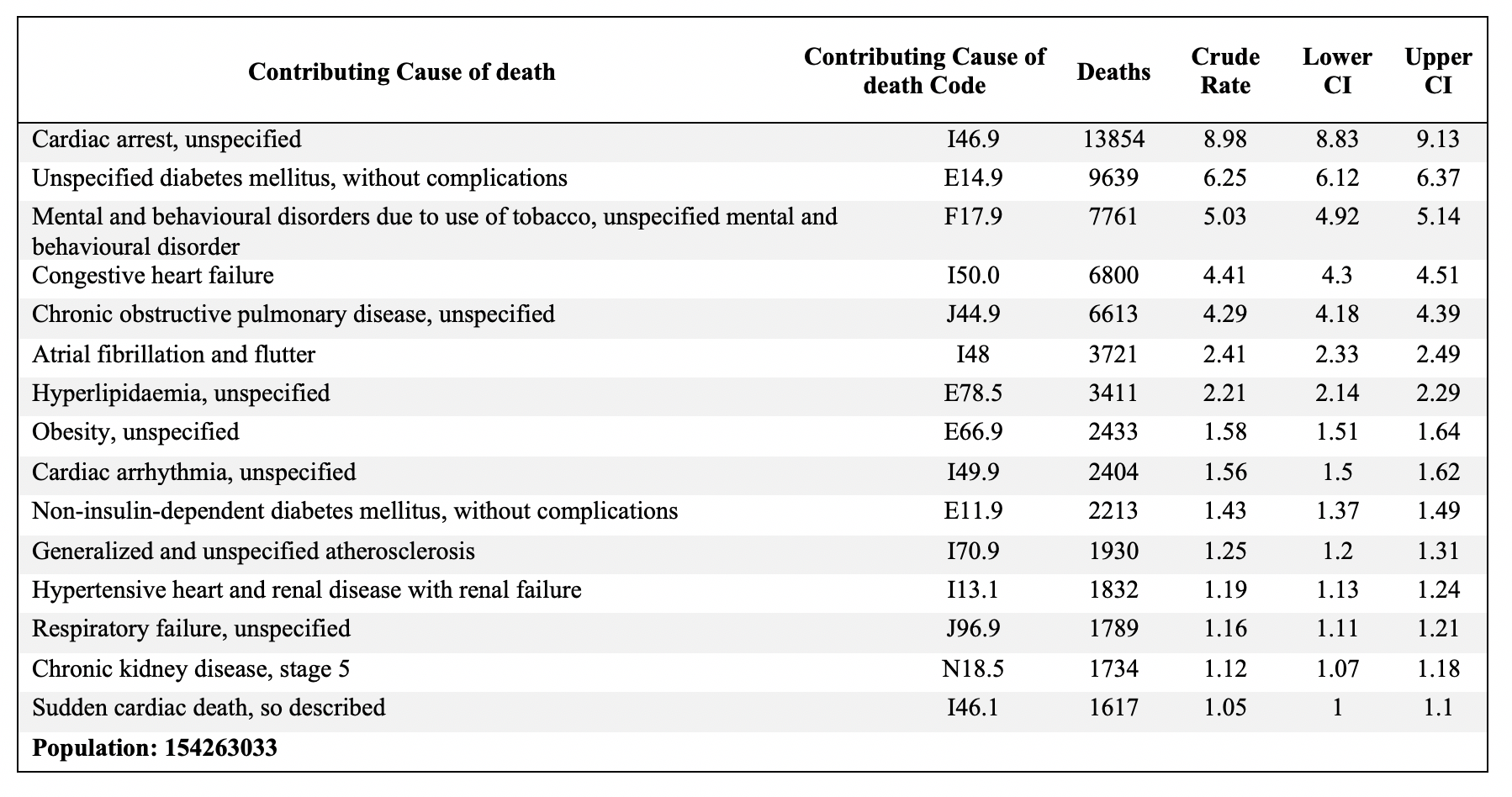

Supplement: S5 Fig — (PNG) [file pone.0225207.s005.png]

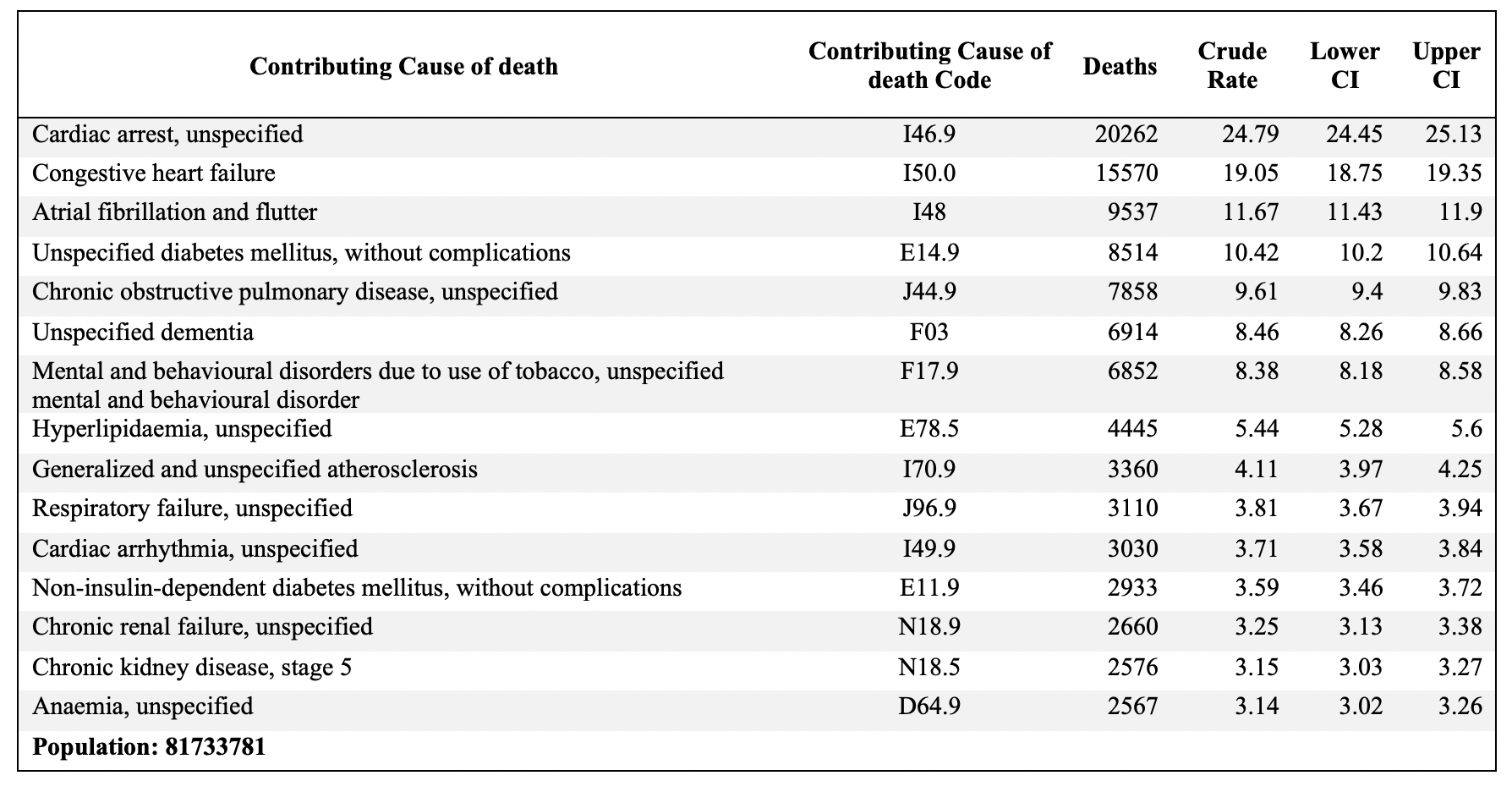

Supplement: S6 Fig — (PNG) [file pone.0225207.s006.png]

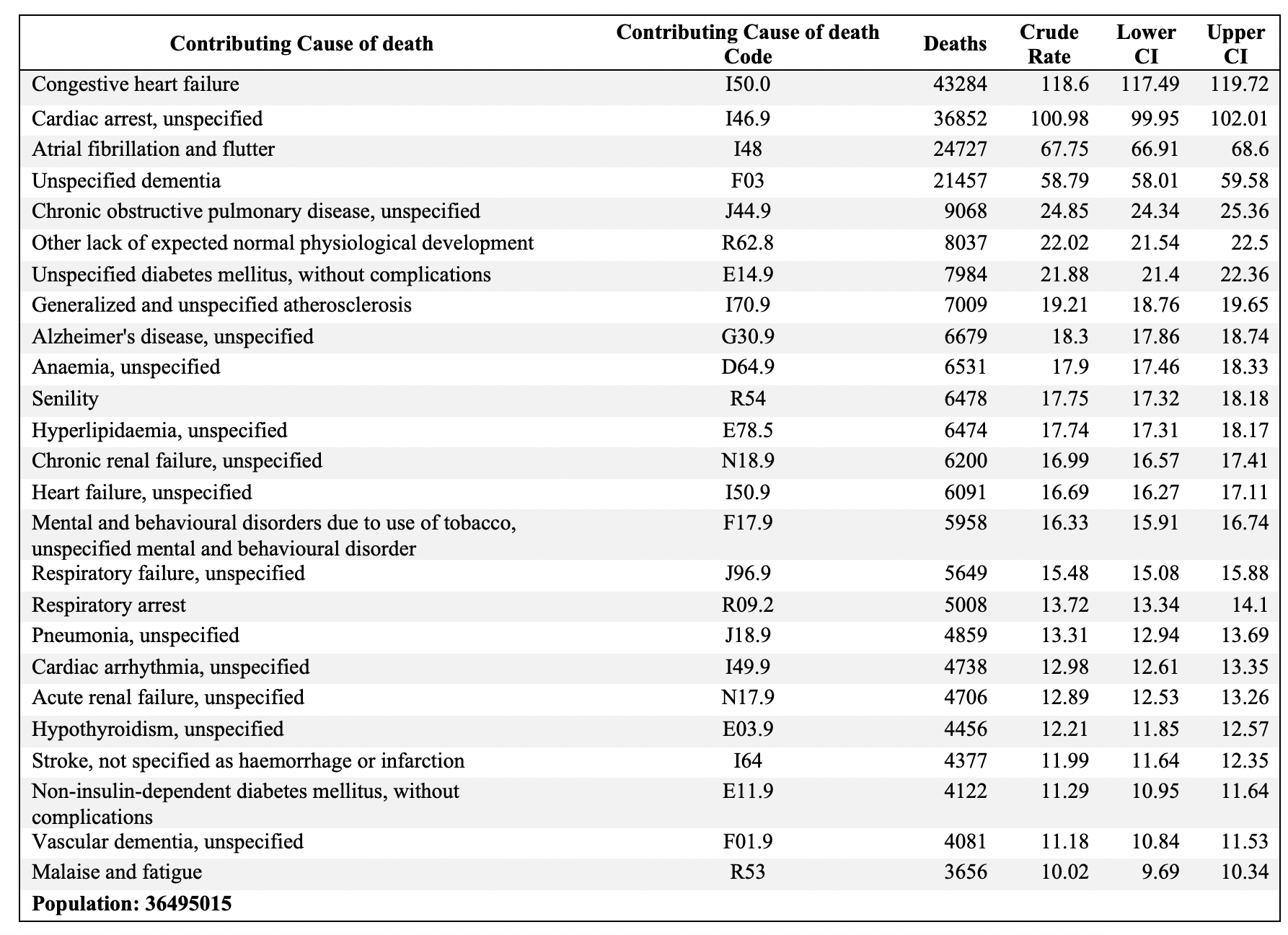

Supplement: S7 Fig — (PNG) [file pone.0225207.s007.png]

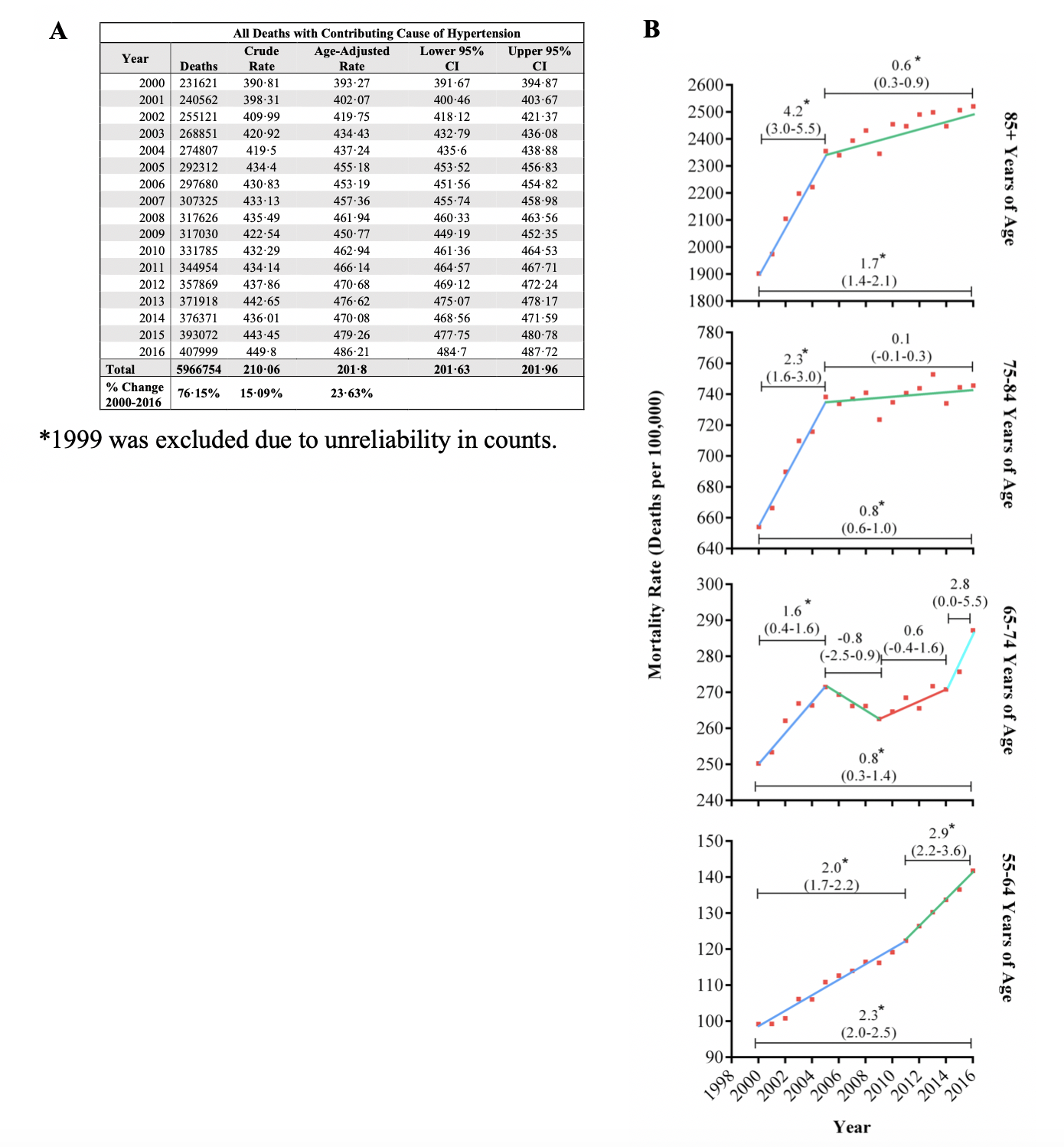

Supplement: S8 Fig — Mortality data were collected on all underlying causes with a contributing cause of hypertension from 2000–2016. (A). Data were then stratified by age group (55–64, 65–74, 75–84, and 85+) and joinpoint analyses were performed (B). Although all ages groups exhibited significant increases over the entire 2000–2016 range, individuals aged 55–74 exhibited increases in their rate starting in the late 2000’s. (PNG) [file pone.0225207.s008.png]

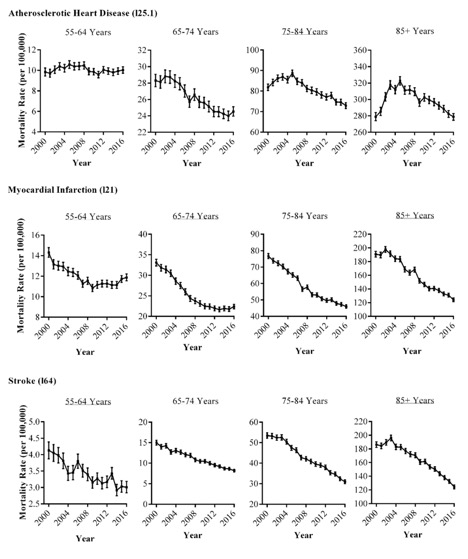

Supplement: S9 Fig — Mortality data were collected on all underlying causes with a contributing cause of hypertension and stratified by age. Data were collected on atherosclerotic heart disease, myocardial infarction, and stroke. ICD-10 codes are listed in parentheses next to disease name. 1999 was excluded from analysis due to un-reliable mortality rates for a few diseases. (JPG) [file pone.0225207.s009.jpg]

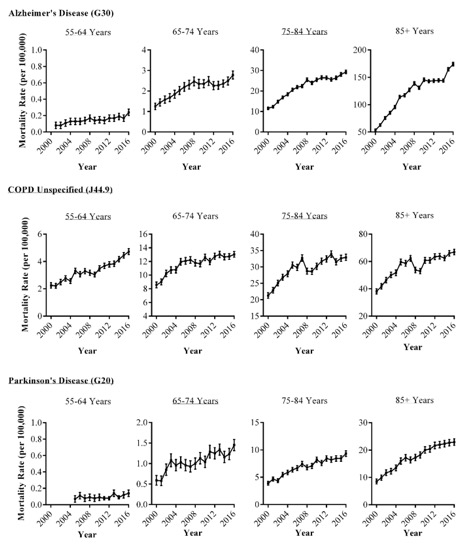

Supplement: S10 Fig — Mortality data were collected on all underlying causes with a contributing cause of hypertension and stratified by age. Data were collected on Alzheimer’s disease, COPD unspecified and Parkinson’s disease. ICD-10 codes are listed in parentheses next to disease name. 1999 was excluded from analysis due to un-reliable mortality rates for a few diseases. (JPG) [file pone.0225207.s010.jpg]

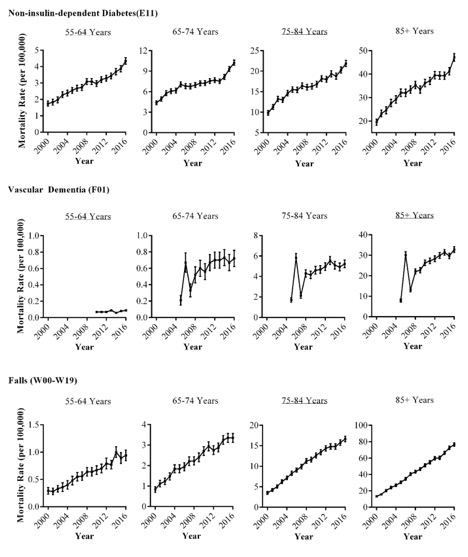

Supplement: S11 Fig — Mortality data were collected on all underlying causes with a contributing cause of hypertension and stratified by age. Data were collected on non-insulin-dependent diabetes, vascular dementia, and falls. ICD-10 codes are listed in parentheses next to disease name. 1999 was excluded from analysis due to un-reliable mortality rates for a few diseases. (JPG) [file pone.0225207.s011.jpg]

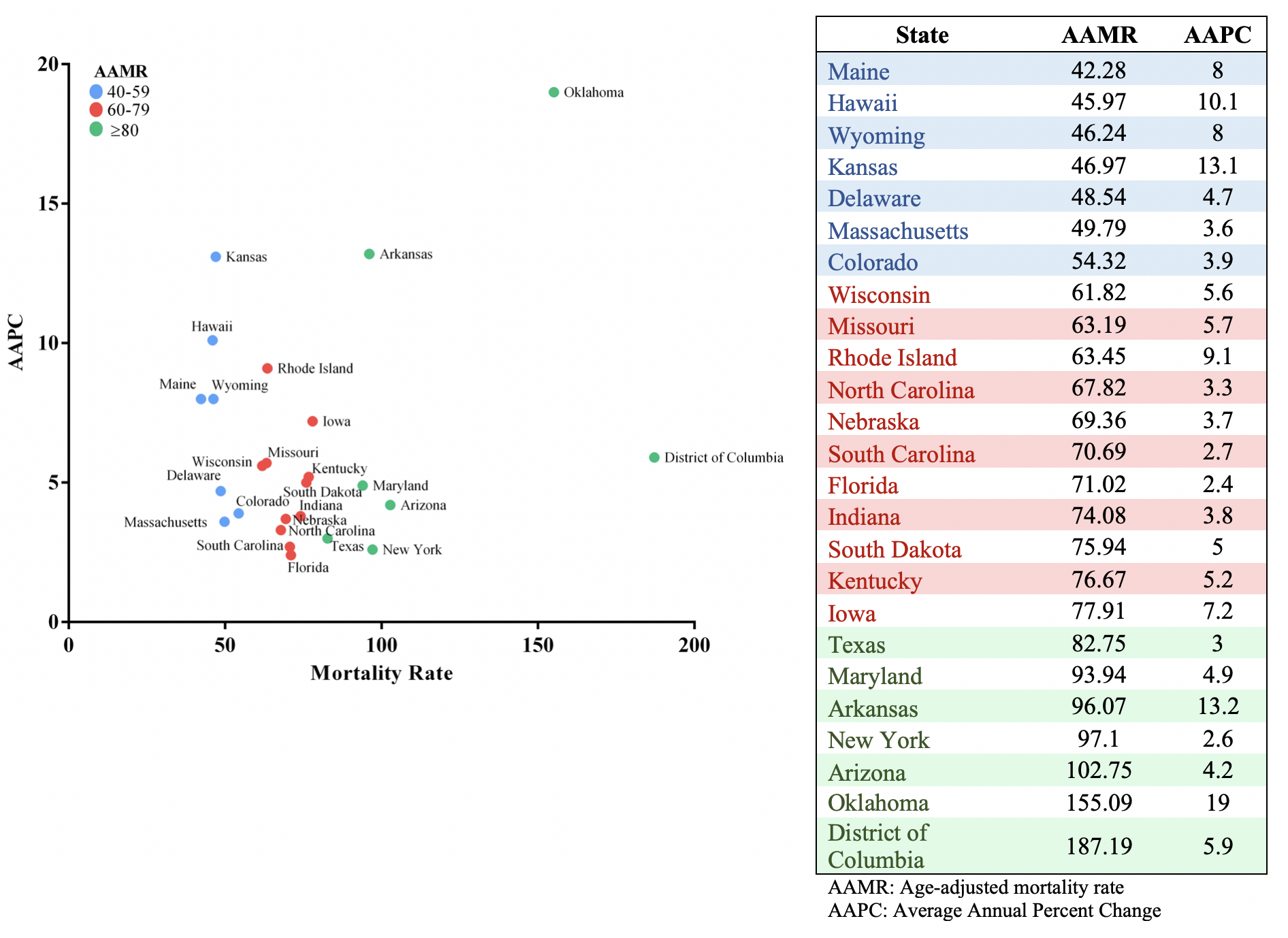

Supplement: S12 Fig — Statistically significant State AAPC for 2011–2018 was plotted with cumulative State age-adjusted hypertension-related mortality data for the years 2011–2016 for individuals 55 years of age and above. (PNG) [file pone.0225207.s012.png]

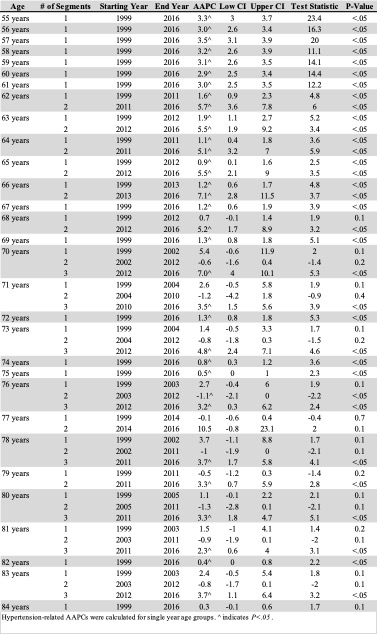

Supplement: S13 Fig — (JPG) [file pone.0225207.s013.jpg]
