# Supplementary material for: An acceleration in hypertension-related mortality for middle-aged and older Americans, 1999-2016: An observational study
Source: PLoS One. 2020 Jan 15;15(1):e0225207. doi: 10.1371/journal.pone.0225207 (PMC6961854; doi:10.1371/journal.pone.0225207)
Supplement: S1 Supplemental Search Term File — (DOCX) [file pone.0225207.s014.docx]

**Search Terms Used in the Study (in no particular order):**

Please see methods/results to see how terms were used for analysis

**1. Organize table layout:**

Group results By: Year, Race, Gender, Hispanic Origin, Census Region, State

Measures: Age adjusted rates with standard error

Additional rate options: 2000 std. population

Non-standard populations: 2000 estimate, 2016 estimate

**2. Select location**: *All*

**3. Select demographics:**

Ten-year age groups: 25-34 through 85+ years

Gender: All genders

Hispanic origin: Hispanic or Latino, Not Hispanic or Latino

Race: All races

**4. Select year and month:** *All*

**5. Select weekday, autopsy, and place of death:**

Weekday: All weekdays

Autopsy: All values

Place of death: All places

**6. Select cause of death:**

Detailed mortality dataset: ICD-10 Codes (See methods for specific codes used)

| **Code** | **Cause** |
| --- | --- |
| I10 | Essential hypertension |
| I11 | Hypertensive heart disease |
| I12 | Hypertensive renal disease |
| I13 | Hypertensive heart and renal disease |
| I15 | Secondary hypertension |

Multiple cause dataset: ICD-10 Codes (See methods for specific combination of codes used)

Contributing cause with underlying hypertension codes:

| **Code** | **Cause** |
| --- | --- |
| E11 | Non-insulin-dependent diabetes mellitus |
| E66 | Obesity |
| FO1 | Dementia |
| I46 | Cardiac arrest |
| I48 | Atrial fibrillation |
| I50 | Heart failure |
| J44.9 | COPD, unspecified |

Underlying cause with contributing cause hypertension codes:

| **Code** | **Cause** |
| --- | --- |
| E11 | Non-insulin-dependent diabetes |
| FO1 | Vascular dementia |
| G20 | Parkinson’s disease |
| G30 | Alzheimer’s disease |
| I21 | Myocardial infarction |
| I25.1 | Atherosclerotic heart disease |
| I64 | Stroke |
| J44.9 | COPD, unspecified |
| W00-W19 | Falls |

**7. Other options:** Export results, Show zero values, Precision 2 decimal places
